# Mannose-Binding Lectin (MBL) and MBL-associated serine protease-2 (MASP-2) in women with malignant and benign ovarian tumours

**DOI:** 10.1007/s00262-014-1579-y

**Published:** 2014-07-20

**Authors:** Anna St. Swierzko, Agnieszka Szala, Sambor Sawicki, Janusz Szemraj, Marcin Sniadecki, Anna Sokolowska, Andrzej Kaluzynski, Dariusz Wydra, Maciej Cedzynski

**Affiliations:** 1 Laboratory of Immunobiology of Infections, Institute of Medical Biology, Polish Academy of Sciences, Lodowa 106, 93-232 Lodz, Poland; 2 Department of Gynaecology, Oncologic Gynaecology and Gynaecologic Endocrinology, Medical University of Gdansk, Kliniczna 1a, 80-402 Gdańsk, Poland; 3 Department of Biochemistry, Medical University of Lodz, Mazowiecka 6/8, 92-215 Lodz, Poland; 4 Department of Clinical Pathomorphology, Polish Mother’s Memorial Hospital Research Institute, Rzgowska 281/289, 93-338 Lodz, Poland

**Keywords:** Ovarian cancer, Benign ovarian tumours, Mannose-Binding Lectin (MBL), MASP, Complement

## Abstract

Mannose-Binding Lectin (MBL) is a serum pattern recognition molecule, able to activate complement in association with MASP proteases. Serum levels of MBL and MASP-2, activities of MBL–MASP complexes, single nucleotide polymorphisms of the *MBL2* and *MASP2* genes and/or their specific mRNA expression in ovarian sections were investigated in 128 patients suffering from primary ovarian cancer (OC) and compared with 197 controls (C), encompassing both patients with benign ovarian tumours (*n* = 123) and others with no ovarian pathology (*n* = 74). MBL deficiency-associated genotypes were more common among OC patients than among controls. The *O*/*O* group of genotypes was associated with ovarian cancer (OR 3.5, *p* = 0.02). In *A*/*A* homozygotes, MBL concentrations and activities were elevated in the OC group and correlated with C-reactive protein. Moreover, high MBL serum levels were associated with more advanced disease stage. No differences in distribution of the *MASP2* +*359 A*>*G* (*D120G*) SNP or MASP-2 serum levels were found between cancer patients and their controls. However, the highest frequency of the *A*/*G* (*MASP2*) and *LXA*/*O* or *O*/*O* (*MBL2*) genotypes was found among OC patients with tumours of G1–2 grade (well/moderately differentiated). Furthermore, MBL deficiency-associated genotypes predicted prolonged survival. None of the parameters investigated correlated with CA125 antigen or patients’ age. The local expression of *MBL2* and *MASP2* genes was higher in women with ovarian cancer compared with controls. It is concluded that the expression of MBL and MASP-2 is altered in ovarian cancer, possibly indicating involvement of the lectin pathway of complement activation in the disease.

**Electronic supplementary material:**

The online version of this article (doi:10.1007/s00262-014-1579-y) contains supplementary material, which is available to authorized users.

## Introduction

The complement system may play a dual role in the pathogenesis of cancer. On the one hand, it may contribute to the clearance of potentially tumourigenic cells harbouring pathogen- or danger-associated molecular patterns (PAMPs or DAMPs). On the other hand, complement activity may favour tumour development. For example, it has been suggested (based on an animal model) that myeloid-derived suppressor cells (MDSC), recruited in a C5a-dependent way, stimulate production of reactive oxygen species and reactive nitrogen species (ROS and RNS) and then contribute to the inhibition of a CD8+ T cell-mediated anti-tumour response [1].

Mannose-Binding Lectin (MBL) may interact directly with neoplastic cells (e.g. by MBL-dependent cell-mediated cytotoxicity [2]), or inhibit metalloproteases that degrade extracellular matrix, or protect against chemotherapy-related infections or infections with cancerogenic agents (reviewed by Swierzko et al. [3]). Moreover, MBL may interact with antigen-presenting cells (often present in the tumour microenvironment), influence their activity/proliferation and thereby contribute to the outcome of the anti-tumour immune response [4, 5].

The gene responsible for MBL synthesis (*MBL2*) is localized to chromosome 10 (10q11.2–q21). Single nucleotide polymorphisms (SNPs) in its first exon are responsible for altered MBL serum concentration and impaired function. The dominant alleles *D*, *B* and *C* (collectively designated *O*), corresponding to mutations in codons 52, 54 and 57, respectively, are associated with lower MBL levels compared with the *A* (wild-type or normal) allele. Polymorphisms in the promoter region (*H*/*L* and *Y*/*X* at positions −550 and −221, respectively) also influence the serum protein concentration. Homozygotes or compound heterozygotes for variant alleles (*O*/*O*) as well as *LXA*/*O* heterozygotes are considered to be MBL deficient (reviewed in [3]). Although MBL is predominantly synthesized in the liver, some *MBL2* gene expression (at the mRNA level) has been found in bone marrow, fetal lung, small intestine and testis [6]. We ourselves have reported the presence of MBL protein and specific mRNA in both normal and malignant ovaries. Moreover, MBL protein was detected in ascites from women with ovarian cancer [7]. The transcription of the *MBL2* gene is known to be up-regulated by IL-6, dexamethasone, heat shock, thyroid hormones and growth hormone while down-regulated by IL-1 [8, 9].

The ability of MBL to activate the complement cascade results from its cooperation with MBL-associated serine proteases (MASPs), encoded by the *MASP1*/*3* and *MASP2* genes, localized to chromosomes 3 (3q27–q28) and 1 (1p36.2–3), respectively [10]. MASP-2 cleaves complement factors C4 and C2 with high efficiency. Among Caucasians, one SNP (+*359 A*>*G*; *D120G*) leads to diminished MASP-2 activity in heterozygotes and total MASP-2 deficiency in homozygotes. This mutation affects the structure of the CUB1 domain and abolishes interaction with the pattern recognition molecules of the lectin pathway of complement activation [11].

MASP-1 was demonstrated to associate with MASP-2 in the same complex with MBL and to activate MASP-2 directly. Moreover, it is able to activate factor C2, producing the majority (60 %) of C2a molecules, necessary for the C3 convertase. Thus, it is considered to be crucial for lectin pathway activation [12–14]. MASP-1 could possibly also be involved in the coagulation cascade with its substrates being fibrinogen, factor XIII and thrombin-activatable fibrinolysis inhibitor (TAFI). Moreover, its thrombin-like activity enables cleaving of protease-activated receptor-4, a mediator of inflammation and platelet activation (reviewed by Yongqing et al. [15] and Matsushita et al. [16]). Recently, Dobo et al. [17] found evidence that another MASP-1 substrate is high-molecular-weight kininogen. This activity (like that of kallikrein) enables release of bradykinin, a highly pro-inflammatory mediator of the contact system. Although these authors noted that MASP-2 also cleaved kininogen, no bradykinin was released. To date, no case of MASP-1 deficiency has been described, but curiously, fish and birds do well without possessing MASP-1 [18, 19].

The liver is the main source of both MASP-1 and MASP-2, but low expression at the mRNA level has also been found in colon, heart, lung, kidney, placenta, brain (for MASP-1), and in testis and small intestine (for both MASP-1 and MASP-2) [20]. In contrast to the *MBL2* gene, transcription of *MASP1*/*3* and *MASP2* was shown to be stimulated by IL-1β and abolished by IL-6. Moreover, *MASP1*/*3* expression is down-regulated by IFN-γ [21].

We previously reported an association between low MBL-conferring haplotypes and ovarian cancer, but surprisingly no corresponding relationship with serum MBL concentration or activity [7]. Also, *MBL2* gene expression was detected in all ovarian tissues examined, but significant *MASP2* expression was confined to malignant ovarian tissue only. We later found elevated serum Ficolin-2 (L-ficolin) and Ficolin-3 (H-ficolin) in ovarian cancer patients, while the local expression of the corresponding genes was decreased [22]. In the present case-controlled, retrospective study, we have revisited the relationship between MBL and ovarian cancer with an entirely separate series of patients and controls in order to confirm or refute the apparent paradox (association with genetic MBL deficiency, but not serum MBL deficiency) reported earlier, and we have supplemented our investigations to include MASP-2 concentrations, *MASP2* genotyping and MBL–MASP-1 complex activities. Additionally, gene expression data have been markedly extended.

## Materials and methods

### Patients and controls

Both cancer patients and their controls attended the Department of Gynaecology, Oncologic Gynaecology and Gynaecologic Endocrinology, Medical University of Gdansk, Poland and were identical to the patient groups described in our Ficolin-2 and Ficolin-3 study [22]. A total of 128 women (aged 28–86 years, mean: 58.6) had the diagnosis of primary ovarian cancer (OC group). The majority of those patients suffered from serous carcinoma (*n* = 83), but twenty-five patients had ovarian tumours of other histological types, including endometrioid, mucinous and clear-cell carcinomas. Seventy-eight patients had advanced disease stage (FIGO stage III–IV) while 25 were classified as FIGO I–II. Sixty-five women had poorly differentiated tumours (G3), while 33 had well-differentiated tumours (G1–2). For some patients, complete clinical data were not available. Ovarian cancer patients were compared with two separate reference groups of non-cancer patients, collectively classified as controls (C): 123 patients diagnosed with benign tumours of the ovary (BT; aged 19–82 years, mean: 45.6) and 74 patients operated on because of leiomyomas or dysfunctional uterine bleeding but without pathological changes in the ovaries (NO group; aged 20–76 years, mean: 48.2). The BT group included patients with ovarian serous cysts, adenomas, fibromas, teratomas or endometriosis. Blood from all patients was taken preoperatively while tissue samples were taken during primary surgery. Approval of the local ethical committee was obtained, as was the written informed consent of patients.

### Blood, serum and ovarian tissue samples

Blood samples for DNA preparation were taken into tubes containing sodium citrate and stored at −20 °C. Sera were prepared from blood samples collected into tubes without anticoagulant and stored at −70 °C until testing. Tissue samples were collected into tubes with RNALater (Life Technologies, USA) and stored at −70 °C.

### Determination of protein concentrations and activities

Serum MBL concentrations [23], MBL–MASP-2 complex activities [23] and MASP-2 concentrations [24] were measured by ELISA as described previously. Murine anti-human MBL mAbs (clone HYB 131-01; BioPorto, Denmark), goat polyclonal anti-human C4 Abs (Calbiochem, USA) and corresponding HRP-conjugated secondary antibodies were used for detection of MBL and activated C4b fragment (for an estimation of MBL–MASP-2 complex activity), respectively. In the case of MASP-2 concentration (“sandwich ELISA”), rat anti-MASP-2 mAb (clone 8B5) was used for coating while biotinylated rat anti-MASP-2/MAp19 mAb (clone 6G12) followed by Eu^3+^-labelled streptavidin (Perkin Elmer, USA)—for detection. Both antibodies were kindly provided by Prof. Jens C. Jensenius (Aarhus University, Denmark). To test MBL–MASP-1 complex activity, the fluorescence method described by Presanis et al. [25] was used. Briefly, sera prediluted 1:10 were added to mannan-coated wells of microfluor white plates (NUNC, Denmark). VPR-AMC peptide (Bachem, Switzerland) was used as the substrate for MASP-1. The samples were excited at 355 nm, and emission was read at 460 nm every 30 s for 1 h, using a Varioskan Flash reader (Thermo Scientific, USA). Serum from a healthy volunteer (containing 2,000 ng MBL/ml, with *YA*/*YA* *MBL2* genotype) was used as a standard (MBL–MASP-1 complex activity in this serum was arbitrarily assigned the value of 1 U/ml).

In general, “low values” corresponded to below the 10th percentile (MASP-2 concentration) among controls or the detection limits (MBL–MASP-1 and MBL–MASP-2 activities) and “high values” to above the 90th percentile among controls (chosen arbitrarily). An exception was made for MBL (10th percentile: 55 ng/ml) deficiency, as the value of 100 ng/ml appears to be widely accepted [26, 27]. Consequently, a cut-off for “high MBL” (3,000 ng/ml) exceeded the 90th percentile (1,998 ng/ml) within the control group. Detection limits were as follows: 10 ng/ml (MBL concentration), 25 ng/ml (MASP-2 concentration), 60 mU/ml (MBL–MASP-2 activity); and 50 mU/ml (MBL–MASP-1 activity). Values below those mentioned above were displayed as “1” in logarithmic-scale graphs.

### Investigation of single nucleotide polymorphisms of the *MBL2* and *MASP2* genes

DNA was extracted from blood samples with the use of GeneMATRIX Quick Blood Purification Kit (EURx Ltd, Poland), according to the manufacturer’s protocol. Single nucleotide polymorphisms of the *MBL2* [28] and *MASP2* [24] genes were analysed as previously described. The *MBL2* genotypes: *O*/*O* (where “*O*” corresponds to *B*, *C* or *D* exon 1 mutations) and *LXA*/*O* (where “L” and “X” correspond to the promoter variants at positions −550 and −221, respectively while “*A*” to wild-type exon 1) were considered to define MBL deficiency.

### Determination of *MBL2* and *MASP2* gene expression using real-time PCR method

The *MBL2* and *MASP2* gene expression levels were investigated essentially as previously described [7]. PCR primers specific for TATA-box protein (TBP, used as a housekeeping gene) were as described by Li et al. [29].

Analyses were performed with ABI Prism 7700 (SDS Software). Results were normalized to values obtained for TBP. Relative gene expression levels were obtained using the ∆∆*C*_t_ method [30]. Specificity of amplification was further confirmed by obtaining melting curve profiles.

### Statistical analysis

The Statistica (version 10, StatSoft Poland) software package was used for data management and statistical calculations. Median serum MBL and MASP-2 concentrations, activities of MASP-1 and MASP-2, complexed with MBL as well as *MBL2* and *MASP2* gene expressions in ovarian sections were compared by the Mann–Whitney *U* test. Correlations were determined by Spearman’s test. The frequencies of *MBL2* and *MASP2* gene variants were compared by Fisher’s exact test (two-sided). Odds ratios were obtained with the use of MediCalc software (http://www.medcalc.org).

Deviations of the observed genotype frequencies from Hardy–Weinberg equilibrium were calculated separately for the C and OC groups. An exact test was applied, due to the low frequency of certain gene variants [31]. For the *MBL2* gene, data for each exon 1 SNP were calculated separately (*D*, *B* and *C*). The 36-month survival was calculated using the Kaplan–Meier method, and the groups were compared with the use of a log-rank test. *P* values <0.05 were considered statistically significant.

## Results

### Polymorphisms of *MBL2* and *MASP2* genes

Variant alleles of the *MBL2* structural gene were found in 46 (39 %) of 117 OC patients and in 60 (35 %) of 172 controls. Within the C group, these variants were commoner among women with benign ovarian tumours (Table 1). However, *O*/*O* genotypes were significantly more frequent in OC patients than in controls (9.4 vs 2.9 %, *p* = 0.03; Table 1) and associated with an increased probability of developing ovarian cancer (OR 3.5; *p* = 0.02). Taking into account promoter polymorphisms, the higher frequency of MBL deficiency-associated genotypes (*LXA*/*O* + *O*/*O*) among OC patients (18.8 vs 11.7 %; OR 1.8; *p* = 0.09 when compared with C group) reflected a difference from the NO reference group alone (18.8 vs 6.2 %; OR 3.5; *p* = 0.03) (Table 1). Frequencies of *A* (wild-type), *B*, *C* and *D* alleles in each group are given in Supplementary Table 1. The *D* allele (codon 52 mutation) was more frequent in both tumour (BT and OC) groups relative to the NO group. No significant deviation from Hardy–Weinberg equilibrium was found for polymorphisms at codons 52 (*A*/*D*), 54 (*A*/*B*) or 57 (*A*/*C*) (*p* > 0.05).

**Table 1:** Frequency of *MBL2* (**a**) and *MASP2* (b) genotypes in ovarian cancer patients and controls

*MBL2* gene	Group (*n*)			
	C (172)	NO (65)	BT (107)	OC (117)
(a)				
*A*/*A*	65.1	69.2	62.6	60.7
*YA*/*O*	23.3	24.6	22.4	20.5
*XA*/*O*	8.7	4.6	11.2	9.4
*O*/*O*	2.9	1.5	3.7	9.4^a^
*XA*/*O* + *O*/*O*	11.6	6.2	15	18.8^b^

*MASP2* gene	Group (*n*)			
	C (174)	NO (67)	BT (107)	OC (117)
(b)				
*A*/*A*	91.4	92.5	90.7	87.2
*A*/*G*	8.6	7.5	9.3	12.8

C, control group, including NO, patients with no ovarian pathology and BT, patients with benign ovarian tumours; OC, patients with primary ovarian cancer

^a^ *p* = 0.03; OR 3.5 (vs C)

^b^ *p* = 0.03; OR 3.5 (vs NO)

No variant homozygote for +*359 A*>*G* (*D120G*) *MASP2* gene polymorphism was found. Although a trend towards higher frequency of heterozygosity in the OC group was observed (Table 1), the differences in comparison with C, NO or BT groups did not reach statistical significance. No significant deviation from Hardy–Weinberg equilibrium was observed (*p* > 0.05).

**Fig. 1:**
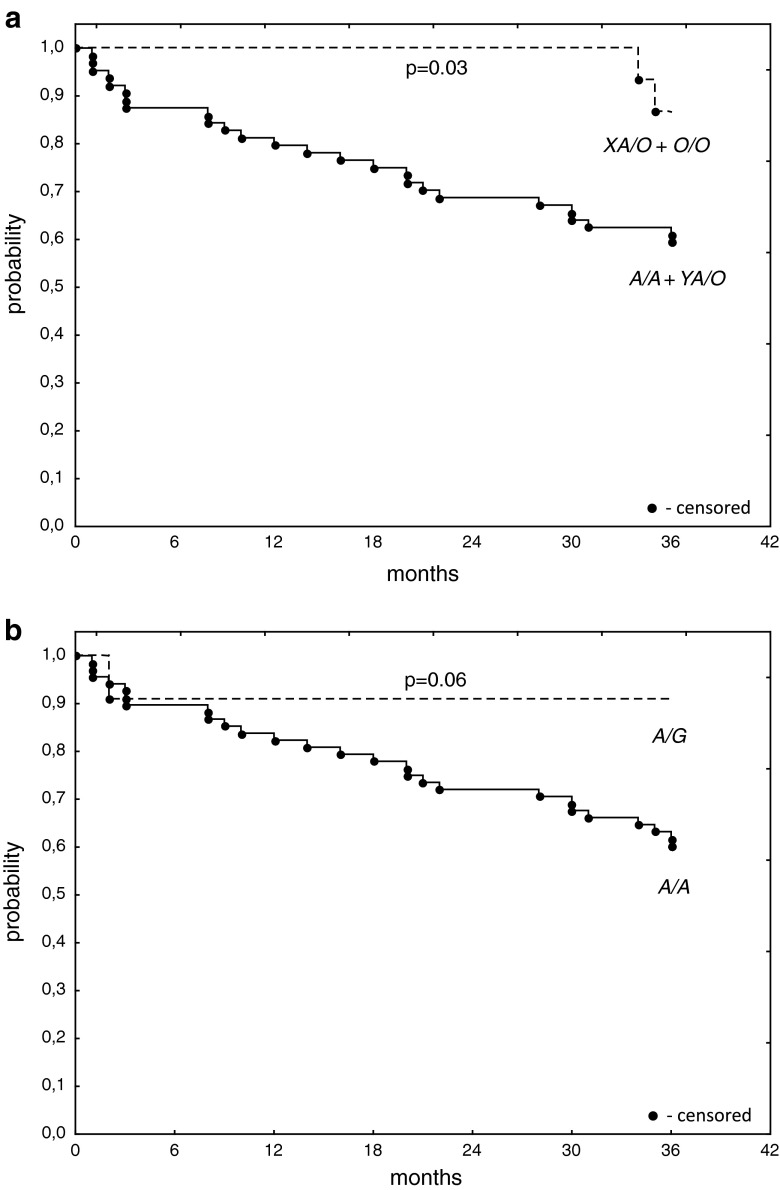
Kaplan–Meier plot of *MBL2* (**a**) and *MASP2* (**b**) genotypes and survival in patients with ovarian cancer

Interestingly, the frequencies of both MBL deficiency-associated genotypes (*LXA*/*O* + *O*/*O*) and *MASP2* *A*/*G* heterozygosity were highest among patients with well-/moderately differentiated tumours (G1–2 grade) (Table 2). Analysis of survival of OC patients (*n* = 79) performed three years after surgery revealed better survival of carriers of *MBL2 LXA*/*O* or *O*/*O* (*n* = 15) compared with *A*/*A* or *YA*/*O* genotypes (log-rank test: *p* = 0.03; Fig. 1a). For *MASP2* gene, the corresponding trend did not quite reach statistical significance (*p* = 0.06, Fig. 1b). Nevertheless, 10 out of 51 patients who survived ≥36 months carried the *D120G* mutation (compared with 1/28 of those patients who died within 3 years after operation).

**Table 2:** Frequency of MBL deficiency-associated genotypes and *MASP2 A*/*G* heterozygosity

Group	C	NO	BT	OC G1-2	OC G3
*n*	172 (174)^a^	65 (67)^a^	107	33	63
MBL deficiency-associated genotypes (*LXA*/*O* + *O*/*O*) (%)	11.6	6.2	15	30.3^b^	12.7
*MASP2 A*/*G* heterozygotes (%)	8.6	7.5	9.3	24.2^c^	7.9
*LXA*/*O* + *O*/*O* (*MBL2*) and *A*/*G* (*MASP2*) (%)	0.6	0	0.9	12.1^d^	0

C, control group, including NO, patients with no ovarian pathology and BT, patients with benign ovarian tumours; OC, patients with primary ovarian cancer

^a^Numbers of samples tested for D120G *MASP2* polymorphism

^b^ *p* = 0.05 (vs OC G3); *p* = 0.01 (vs C); *p* = 0.04 (vs NO); *p* = 0.07 (vs BT)

^c^ *p* = 0.06 (vs OC G3); *p* = 0.02 (vs C); *p* = 0.03 (vs NO); *p* = 0.04 (vs BT)

^d^ *p* = 0.01 (vs OC G3); *p* = 0.002 (vs C); *p* = 0.01 (vs NO); *p* = 0.01 (vs BT)

### Serum MBL and MASP-2 concentrations

**Fig. 2:**
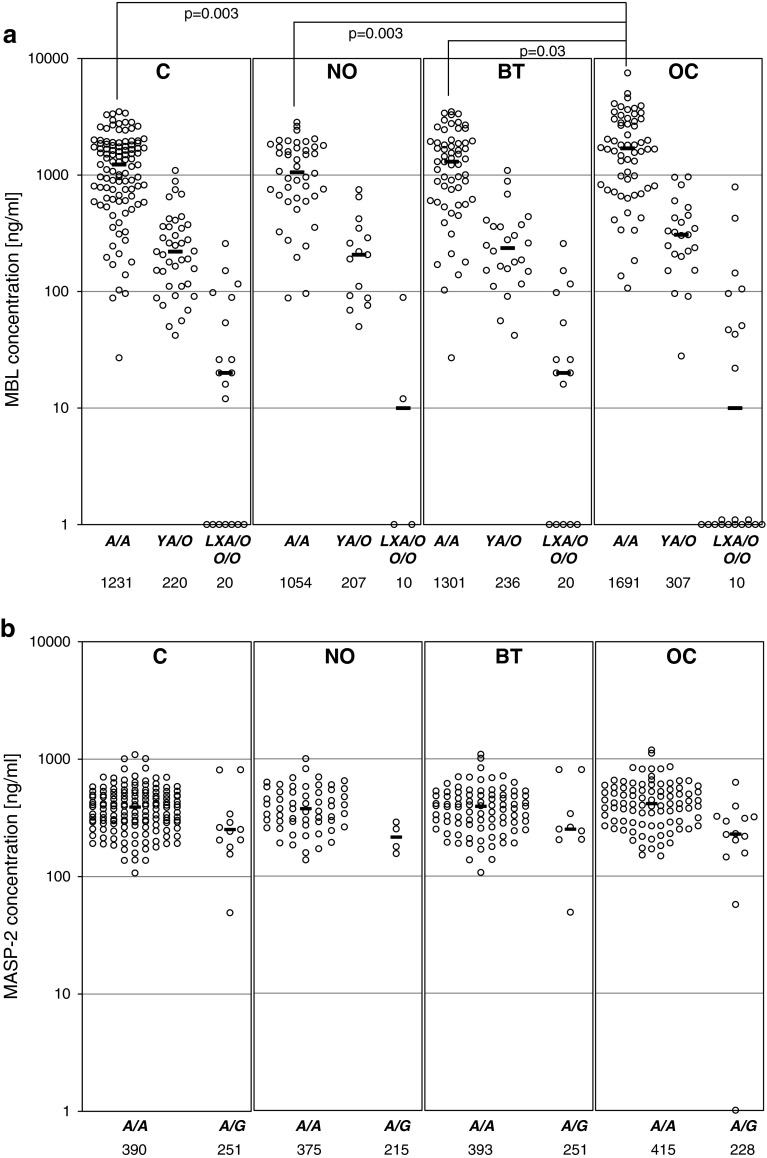

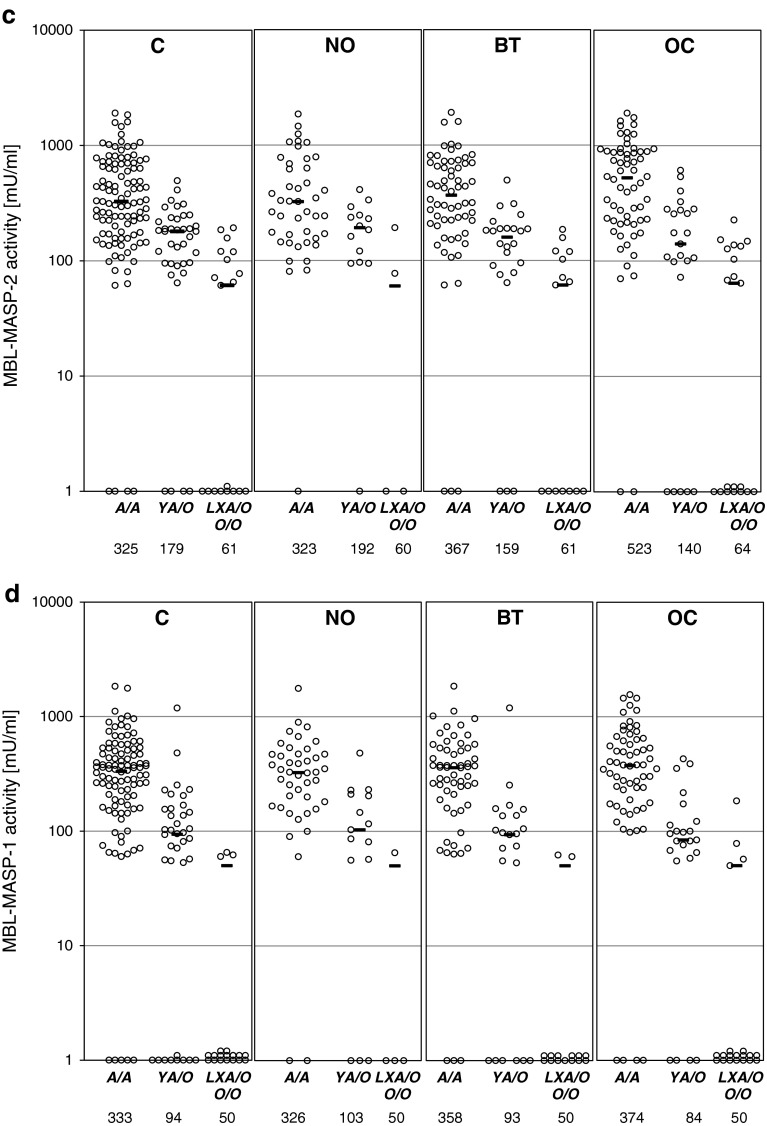
**a** Individual MBL serum concentrations, depending on *MBL2* genotypes; **b** individual MASP-2 serum concentrations, depending on *MASP2* genotypes; **c** individual MBL–MASP-2 serum activities, depending on *MBL2* genotypes; **d** individual MBL–MASP-1 serum activities, depending on *MBL2* genotypes. C, combined control group; NO, women with normal ovaries; BT, patients with benign ovarian tumours; OC, patients with primary ovarian cancer. Values below the detection limit are presented as “1”.* Bars* indicate median values (presented by numbers below corresponding genotypes). When the majority of values were under the detection limit, medians were marked at that level

The median serum MBL in the OC patients (714 ng/ml) did not differ significantly from that of the control group (641 ng/ml) (full details are presented in Supplementary Table 2) However, *MBL2 A*/*A* homozygotes suffering from ovarian cancer had generally higher MBL concentrations (median 1,692 ng/ml) than their counterparts in the C (1,231 ng/ml), NO (1,054 ng/ml) or BT (1,301 ng/ml) groups (Fig. 2a). Moreover, the highest (>3,000 ng/ml) MBL levels were significantly commoner in OC than in any of the reference groups while no difference in the frequency of the lowest MBL levels (<100 ng/ml) was found (Table 3).

**Table 3:** Incidence of extreme (low or high) concentrations/activities of MBL and MASPs in ovarian cancer patients and controls

Parameter	Cut-off	Frequency in groups			
		C (*n* = 164)^a^	NO (*n* = 62)^b^	BT (*n* = 102)^c^	OC (*n* = 107)^d^
MBL concentration	<100 ng/ml	27 (16.5)	11 (17.7)	16 (15.7)	20 (18.7)
	>3,000 ng/ml	4 (2.4)	0	4 (3.9)	15 (14)^e^
MASP-2 concentration	<191 ng/ml	14 (8.6)	6 (9.7)	8 (8)	9 (8.5)
	>627 ng/ml	17 (10.5)	6 (9.7)	11 (11)	14 (13.2)
MBL–MASP-2 activity	<60 mU/ml	18 (11)	4 (6.6)	14 (13.6)	18 (16.8)
	>800 mU/ml	17 (10.4)	7 (11.5)	10 (9.7)	21 (19.6)^f^
MBL–MASP-1 activity	<50 mU/ml	34 (21)	8 (12.9)	26 (26)	29 (27.1)^g^
	>605 mU/ml	17 (10.5)	6 (9.7)	11 (11)	17 (15.9)

Percentages are given in parentheses

C, control group, including NO, patients with no ovarian pathology and BT, patients with benign ovarian tumours; OC, patients with primary ovarian cancer

^a^ *n* = 162 for MASP-2 concentration and MBL–MASP-1 activity

^b^n = 61 for MBL–MASP-2 activity

^c^n = 100 for MASP-2 concentration and MBL–MASP-1 activity; *n* = 103 for MBL–MASP-2 activity

^d^n = 106 for MASP-2 concentration

^e^ *p* = 0.0004; OR 6.5 (vs C); *p* = 0.001; OR 21 (vs NO); *p* = 0.01; OR 4 (vs BT)

^f^ *p* < 0.05; OR 2.1(vs C)

^g^ *p* = 0.035; OR 2.5 (vs NO)

Histological grading was known for 13 patients with very high (>3,000 ng/ml) serum MBL: 12 were G3 (OR 7.3; *p* = 0.05, vs G1–2 patients). Fifteen very high serum MBL patients were FIGO staged: 13 were grades III or IV (OR 2.1; *p* = 0.5 vs FIGO I–II patients).

In general, MASP-2 concentrations did not differ between the groups (Supplementary Table 2), even when *MASP2* genotype was taken into account (Fig. 2b). Furthermore, the frequencies of extreme levels (<191 ng/ml or >627 ng/ml) were much the same (Table 3).

MBL concentrations correlated inversely with MASP-2 levels in the control patients (*R* = −0.24; *p* = 0.002) but not in the OC group. MBL levels weakly correlated with CRP within the OC group only (*R* = 0.3, *p* < 0.05). No correlations with CA125 antigen concentration or patients’ age were noted (not shown).

### Activities of MBL–MASP complexes

Median MBL–MASP-1 (173 mU/ml) and MBL–MASP-2 (233 mU/ml) activities in the OC patients did not differ significantly from those of their controls (202 and 224 mU/ml, respectively) (Supplementary Table 2), even in *MBL2* *A*/*A* homozygotes (Fig. 2c, d). However, when patients carrying both *MBL2 A*/*A* and *MASP2 A*/*A* genotypes were compared, MBL–MASP-2 activity was highest in the OC group (*p* < 0.05 compared with the C group) (Supplementary Table 3).

Similarly, high values for MBL–MASP-2 activity were commoner in the OC than in the C patients (*p* < 0.05), but the corresponding trend for MBL–MASP-1 activity did not achieve statistical significance (Table 3). Frequencies of functional MBL–MASP-2 relative deficiency (activity <60 mU/ml) did not differ between the groups, but MBL–MASP-1 deficiency (<50 mU/ml) was more common in OC compared with the NO group (*p* = 0.035) (Table 3).

As expected, highly significant correlations between serum MBL and MBL–MASP-1 activity (0.88 ≤ *R* ≤ 0.91) and between serum MBL and MBL–MASP-2 activity (0.73 ≤ *R* ≤ 0.85) were noted in all groups. None of the parameters investigated correlated with CA125 or patients’ age (not shown).

### MBL and MASP-2-specific mRNA expression in ovarian sections

**Fig. 3:**
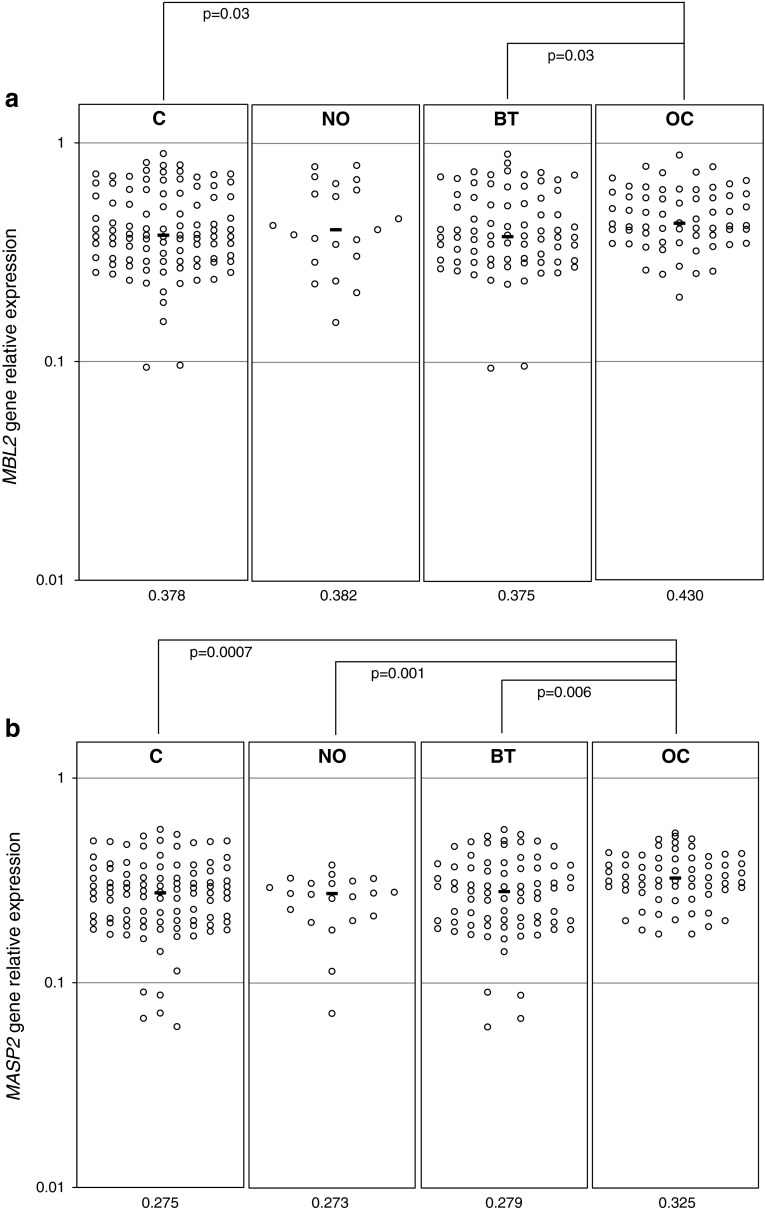
Expression (at the mRNA level) of *MBL2* (**a**) and *MASP2* (**b**) genes in ovarian section samples. C, combined control group; NO, women with normal ovaries; BT, patients with benign ovarian tumours; OC, patients with primary ovarian cancer. Bars indicate median values (presented by numbers below corresponding genotypes)

Expression of both *MBL2* and *MASP2* genes, at the mRNA level, was detected in all ovarian sections tested from OC patients (*n* = 64) and controls (*n* = 103). Median values were significantly higher in OC patients compared with their controls (Fig. 3a, b). For the *MASP2* gene, data from malignant tumours differed significantly from those from benign tumours or normal ovaries (Fig. 3b), but the average relative expression of the *MBL2* gene did not differ between the OC and NO groups (Fig. 3a).

The expression levels of *MBL2* and *MASP2* genes were found to correlate with each other in all groups. Local *MBL2* expression correlated significantly with serum MBL level in controls but not in OC patients. No association between MASP-2-specific mRNA and serum MASP-2 concentration was observed. Moreover, neither *MBL2* nor *MASP2* expression level correlated with CA125 antigen or age of patients (not shown).

## Discussion

Various disorders (endometriosis, pelvic inflammatory disease, etc.) are postulated to increase the risk for epithelial ovarian cancer (reviewed by Maccio and Madeddu [32]). MBL, by participating in the clearance of microorganisms, might help to limit inflammation in the reproductive system. As mentioned, it may also interact directly with certain cancer cells or inhibit extracellular matrix-degrading enzymes. The results reported here broadly confirm our previous finding (from another cohort) [7] that MBL deficiency-associated genotypes are over-represented in ovarian cancer. In contrast to the earlier study, controls (previously defined as “healthy women with no history of cancer”) were recruited on the basis of histopathological examination enabling us to distinguish between patients with benign ovarian tumours and those without any ovarian pathology. The *MBL2* relationship was stronger in comparison with the latter group. Recently, Nevadunsky et al. [33] have also postulated that the *MBL2* gene *B* variant may be a risk factor for ovarian cancer. Several reports demonstrated the significance of *MBL2* gene SNPs in gastric [34, 35], hepatic [36] or colon cancers [37], glioma [38] and acute lymphoblastic leukaemia [39]. In contrast, Ytting et al. [40] found no such association with colorectal cancer.

We also confirmed the absence of association of serum MBL activities (both binding to mannan and complement-activating ability) and ovarian cancer. Indeed, OC patients with normal (wild-type) genotypes had higher levels of serum MBL than their controls. This is likely to be a nonspecific response to an inflammatory stimulus and accounts for the correlation with C-reactive protein. Earlier, elevated MBL concentrations in patients with papillary thyroid carcinoma (compared with subjects with thyroid adenoma and healthy controls) were reported. However, *MBL2* polymorphisms were not analysed in that study [41].

The reason for an association with genetically defined MBL deficiency but not serum protein deficiency is not obvious. One possibility is the existence of a cancer susceptibility gene in linkage disequilibrium with variant *MBL2* alleles, which can account for a number of inconsistent findings in the context of MBL and various malignant and non-malignant diseases (discussed by Kilpatrick [26]). Alternatively, this putative linked gene may act as a disease modifier, accounting for the prediction of longer survival by MBL deficiency-associated (*LXA*/*O*, *O*/*O*) genotypes. Conversely, the association between very high serum MBL and poor prognostic indicators (grade 3 tumours and FIGO stage III-IV) is consistent with this view as all the very high serum MBL patients had wild-type genotypes.

Neither MASP-2 serum concentration nor the +*359 A*>*G* polymorphism of its gene was associated with ovarian cancer. The former correlated with Ficolin-3 levels (reported previously by Szala et al. [22]), within both OC and C groups (not shown). Earlier, Ytting et al. [42, 43] reported high MASP-2 serum level to be a biomarker predicting recurrence and poor survival of patients with colorectal cancer. That was not associated with *MASP2* polymorphism [40]. Moreover, elevated MASP-2 levels in children with tumours of the central nervous system [44] and in patients with papillary thyroid carcinoma [41] have been reported.

An important novel aspect of this study was investigation of local *MBL2* and *MASP2* expression, at the mRNA level. The *MASP2* gene relative expression was significantly higher in malignant ovaries compared with normal organs or those affected by benign tumours. For the *MBL2* gene, the differences were less evident, but a clear difference between OC and BT patients was observed. Interestingly, earlier, we found opposite relationships for Ficolin-2 and Ficolin-3 genes [22]. Consequently, relative expression levels of both *MBL2* and *MASP2* inversely correlated with that of *FCN2* [22] in both OC and C groups (not shown). Recently, expression of the *MBL2* gene in papillary thyroid carcinoma tissue specimens was shown to be higher than in those from adenoma or normal thyroid glands [45].

Since many statistical tests have been performed to compare frequencies of *MBL2* and *MASP2* genotypes/alleles (*MBL2 O*/*O*, *XA*/*O* + *O*/*O* genotypes, *O* alleles; *MASP2 A*/*G* heterozygosity and *G* alleles), protein concentrations/activities (MBL serum levels, MBL–MASP-1 and MBL–MASP-2 activities together with frequencies of extreme values) as well as relative gene expression levels (*MBL2* and *MASP2*), there is a real possibility that some apparently “significant” findings may be due to chance (type 1 error). However, that consideration does not apply to the main conclusions and the statistics underpinning them, because the central findings are confirmations of apparent relationships previously obtained. However, the other relationships indicated here for the first time cannot be considered established, but serve to formulate hypotheses, which may be subsequently confirmed or refuted (ideally by independent researchers).

To summarize, our previous [7, 22] and current data demonstrate certain abnormalities in expression (mRNA, protein levels) of factors specific for the lectin pathway of complement in ovarian cancer. This possibly indicates involvement in pathogenesis, although it is possible that at least some of these disturbances are effects of carcinogenesis. Genetic polymorphisms might influence both risk of disease (*MBL2*) and prognosis (probably *MBL2* and *MASP2*).

## Electronic supplementary material

Below is the link to the electronic supplementary material.

Supplementary material 1 (PDF 171 kb)

## Abbreviations

DAMP: Danger-associated molecular pattern
MASP: Mannose-Binding Lectin-associated serine protease
MBL: Mannose-Binding Lectin
MDSC: Myeloid-derived suppressor cells
PAMP: Pathogen-associated molecular pattern
RNI: Reactive nitrogen species
ROI: Reactive oxygen species
SNP: Single nucleotide polymorphism
