# Supplementary material for: Mannose-Binding Lectin (MBL) and MBL-associated serine protease-2 (MASP-2) in women with malignant and benign ovarian tumours
Source: Cancer Immunol Immunother. 2014 Jul 20;63(11):1129–40. doi: 10.1007/s00262-014-1579-y (PMC4209098; doi:10.1007/s00262-014-1579-y)
Supplement: Supplementary file 1 — Supplementary material 1 (PDF 171 kb) [file 262_2014_1579_MOESM1_ESM.pdf]

Cancer Immunology, Immunotherapy (submitted in 2014) – Anna St. Swierzko et al.

Supplementary Table 1 Frequencies of *MBL2* gene exon 1 alleles (*A*, *B*, *C*, *D*).

Group	C	NO	BT	OC	OC G1-2	OC G3
**n**	172	65	107	117	33	63
A	0.811	0.838	0.794	0.756	0.712	0.770
B	0.131	0.146	0.121	0.171	0.182	0.175
C	0.020	0.008	0.028	0.021	0.030	0.016
D	0.038	0.008	0.056[a]	0.051[b]	0.076[a]	0.040
O	0.189	0.162	0.206	0.244	0.288	0.230

C – control group, including **NO** – patients with no ovarian pathology and **BT** – patients with benign ovarian tumours; **OC** – patients with primary ovarian cancer;
[a] - p=0.02 (*vs* NO)
[b] - p=0.04 (*vs* NO)

Supplementary Table 2 MBL, MASP-2 concentrations and activities of MBL-MASP-2 and MBL-MASP-1 complexes in ovarian cancer patients and controls.

Parameter		Group			
		C (n=164)[a]	NO (n=62)[b]	BT (n=102)[c]	OC (n=107)[d]
MBL concentration [ng/ml]	median	641	708	594	714
	mean	918	894	933	1249
	range	1 – 3493	1 – 2831	1 – 3493	1 – 7524
MASP-2 concentration [ng/ml]	median	375	356	389	394
	mean	398	395	400	420
	range	49 – 1091	137 – 1003	49 – 1091	1 – 1187
MBL-MASP-2 activity [mU/ml]	median	224	242	208	233
	mean	350	374	326	412
	range	1 – 1911	1 – 1848	1 – 1911	1 – 1902
MBL-MASP-1 activity [mU/ml]	median	202	230	158	173
	mean	279	295	270	300
	range	1 – 1839	1 – 1766	1 – 1839	1 – 1555

Detection limits are 10 ng/ml (MBL concentration); 25 ng/ml (MASP-2 concentration); 60 mU/ml (MBL-MASP-2 complex activity); 50 mU/ml (MBL-MASP-1 activity). Values shown as ”1” were under detection limit.
C – control group, including **NO** – patients with no ovarian pathology and **BT** – patients with benign ovarian tumours; **OC** – patients with primary ovarian cancer;
[a] - n=162 for MASP-2 concentration and MBL-MASP-1 activity
[b] - n=61for MBL-MASP-2 activity
[c] - n=100 for MASP-2 concentration and MBL-MASP-1 activity; n=103 for MBL-MASP-2 activity
[d] - n=106 for MASP-2 concentration

Supplementary Table 3 MBL-MASP-2 complex activities in patients carrying A/A (*MBL2*) and A/A (*MASP2*) genotypes.

		Group			
		**C** (n=96)	**NO** (n=39)	**BT** (n=57)	**OC** (n=53)
MBL-MASP-2	median	321	263	337	537[a]
activity	mean	475	458	486	606
[mU/ml]	range	1 – 1911	1 – 1848	1 -1911	1 – 1740

Values shown as ”1” were under detection limit (60 mU/ml)

C – control group, including **NO** – patients with no ovarian pathology and **BT** – patients with benign ovarian tumours; **OC** – patients with primary ovarian cancer;

[a] - p=0.0497 (*vs* C); p=0.07 (*vs* NO); p=0.09 (*vs* BT)
